# Optimal Throughput for Cognitive Radio with Energy Harvesting in Fading Wireless Channel

**DOI:** 10.1155/2014/370658

**Published:** 2014-01-20

**Authors:** Hiep Vu-Van, Insoo Koo

**Affiliations:** School of Electrical Engineering, University of Ulsan, Muger-Dong San-29, Ulsan 680-749, Republic of Korea

## Abstract

Energy resource management is a crucial problem of a device with a finite capacity battery. In this paper, cognitive radio is considered to be a device with an energy harvester that can harvest energy from a non-RF energy resource while performing other actions of cognitive radio. Harvested energy will be stored in a finite capacity battery. At the start of the time slot of cognitive radio, the radio needs to determine if it should remain silent or carry out spectrum sensing based on the idle probability of the primary user and the remaining energy in order to maximize the throughput of the cognitive radio system. In addition, optimal sensing energy and adaptive transmission power control are also investigated in this paper to effectively utilize the limited energy of cognitive radio. Finding an optimal approach is formulated as a partially observable Markov decision process. The simulation results show that the proposed optimal decision scheme outperforms the myopic scheme in which current throughput is only considered when making a decision.

## 1. Introduction

Cognitive radio (CR) technology can improve spectrum utilization by allowing cognitive radio users (CUs) to share the frequency assigned to a licensed user, called the primary user (PU). In order to avoid interference with the operation of the licensed user, CUs are allowed to be active only when the frequency is free. Otherwise, when the presence of the PU is detected, CUs have to vacate their occupied frequency. Subsequently, an essential problem arising in CR implementations is reliable spectrum sensing. In the CR network, since the amount of energy consumed by spectrum sensing increases with sensing time duration, which is one of the main factors affecting sensing performance, sensing energy can significantly affect throughput. In addition, more throughput can be achieved by adapting an adaptive transmission power control (ATPC) [1, 2] in the case of a fading communication channel.

As a normal wireless node, a CU has a finite capacity battery which can be recharged by an energy harvester and is consumed by spectrum sensing, data processing, and data transmission. Therefore, a primary challenge of cognitive radio is how to optimize functionality. The problem of optimal energy management has been considered previously [3, 4] where an optimal energy management scheme for a sensor node with an energy harvester to maximize throughput is proposed. For maximizing throughput of a CR system, the optimal choice about when to keep silent or carry out spectrum sensing is addressed in [5, 6] in which the partially observable Markov decision process (POMDP) [7, 8] is adopted to obtain an optimal secondary access policy. However, in previous works [5, 6] there are some limitations: a constant harvested energy is unrealistic, the effect of energy consumed by performing spectrum sensing on system throughout is not addressed, and an ATPC is not investigated.

In this paper, we propose an optimal mode decision policy (i.e., keep sleeping mode or change to accessing mode) for CR with a non-RF energy harvester to maximize the CR system throughput. An optimal sensing energy algorithm and an ATPC are also considered in the proposed scheme in order to guarantee effective utilization of CU's limited energy resource, which extends life time and improves throughput of the CR system.

## 2. System Model

**Figure 1:**
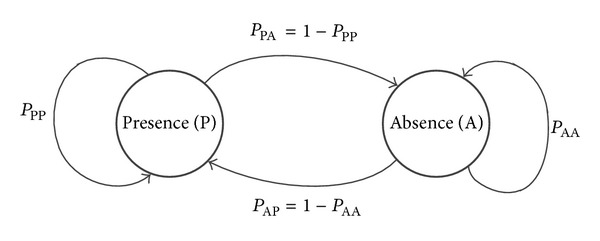
Markov chain states of the PU.

We assume that a CR network and a PU operate in a time slotted model. The status of the PU changes between two states of the Markov chain, that is, presence (P) and absence (A), as shown in Figure 1. The transition probabilities of the PU from state P to state A and from state A to itself are defined as *P*_PA_ and *P*_AA_, respectively. The CU is assumed to always have a data packet to transmit. When the CU wants to access the channel of the PU, it needs to perform spectrum sensing. Only if the sensing result is the state A of PU, CU will be allowed to use the channel.

The energy of the CU is stored in a battery with a finite capacity of *E*_ca_ packets of energy. In general, the CU needs to decide its operation either in sleeping mode or in accessing mode to maximize throughput and energy utilization. In both sleeping and accessing modes, the CU can harvest energy from the environment by using its non-RF harvester while performing other operations. At the *t*th time slot, SU can harvest *E*_*h*_(*t*) energy units that can be used in the next time frame. *E*_*h*_(*t*) takes its value from a finite number *ξ*_*h*_ of energy units:

(1) $$\begin{array}{c} E_{h}\left(t\right)\in \Upsilon_{h}=\left\{e_{1}^{h},e_{2}^{h},\ldots ,e_{\xi_{h}}^{h}\right\}, \end{array}$$

where 0 ≤ *e*_1_^*h*^ < *e*_2_^*h*^ < ⋯<*e*_*ξ*_*h*__^*h*^ ≤ *E*_ca_.

The probability mass function (PMF) of the harvested energy is given as follows:

(2) $$\begin{array}{c} P_{E_{h}}\left(k\right)=\text{Pr}\left[E_{h}\left(t\right)=e_{k}^{h}\right],\;k=1,2,\ldots ,\xi_{h}. \end{array}$$

We assume that the harvested energy follows the stochastic process that is marked by the Poisson process. Subsequently, *E*_*h*_(*t*) is a Poisson random variable with mean *e*_mean_^*h*^. The PMF in (2) can be rewritten as follows:

(3) $$\begin{array}{c} P_{E_{h}}\left(k\right)\approx \frac{e^{-e_{\text{mean}}^{h}}{\left(e_{\text{mean}}^{h}\right)}^{k}}{k!},\;\;k=1,2,\ldots ,\xi_{h}. \end{array}$$

At the beginning of the time frame, information on the amount of remaining energy *e*, 0 ≤ *e* ≤ *E*_ca_ is available at the CU. Furthermore, the CU has a belief *p*, which is the probability of the PU being absent (A) at the time frame. This information can be calculated by statistics of history sensing results from the CR network. Based on the values of *e* and *p*, the SU decides to keep sleeping or to carry out spectrum sensing and transmit data if the state A of the PU is detected.

We consider fading at the data channel between the CU transmitter and the CU receiver. At the CU receiver, we assume that the channel gain takes its value from the set of *L*_*g*_ finite integers:

(4) $$\begin{array}{c} \rho \left(t\right)\in \left\{\rho_{1},\rho_{2},\ldots ,\rho_{L_{g}}\right\}, \end{array}$$

where *ρ*_1_ > *ρ*_2_ > ⋯>*ρ*_*L*_*g*__. The CU receiver reports this channel gain to the CU transmitter over low-rate, error-free, and zero-delay feedback channel, called causal channel state information (CSI) feedback [9].

The PMF of channel gain can be defined as

(5) $$\begin{array}{c} P_{\rho }\left(k\right)=\text{Pr}\left[\rho \left(t\right)=\rho_{k}\right],\;k=1,2,\ldots ,L_{g}. \end{array}$$

By applying an ATPC, the required transmission energy of CU, *E*_*t*_(*t*), can be determined corresponding with the channel gain *ρ*(*t*):

(6) $$\begin{array}{c} E_{t}\left(t\right)\in \Upsilon_{t}=\left\{e_{1}^{t},e_{2}^{t},\ldots ,e_{L_{g}}^{t}\right\}, \end{array}$$

where the smallest required transmission energy, *e*_1_^*t*^, corresponds with the highest channel gain, *ρ*_1_, and, similarly, the CU consumes the largest energy for transmission, *e*_*L*_*g*__^*t*^, when the channel gain is the lowest, *ρ*_*L*_*g*__; that is, *e*_1_^*t*^ < *e*_2_^*t*^ < ⋯<*e*_*L*_*g*__^*t*^ ≤ *E*_ca_. The PMF of transmission energy can be expressed as follows:

(7) PEt(k)=Pr[Et(t)=ekt]=Pr[ρ(t)=ρk], k=1,2,…,Lg.

We assume that the level of channel gain follows the Poisson process. Therefore, *ρ*(*t*) is a Poisson random variable with mean value *ρ*_mean_. As a result, the PMF of the transmission energy in (7) can be given as

(8) PEt(k)=Pr[Et(t)=ekt]=Pr[ρ(t)=ρk]  ≈e−ρmean(ρmean)kk!, k=1,2,…,Lg.

For efficient utilization of energy, we define a transmission energy threshold th_*e*_*t*__ to consider the transmission cost so that if the required transmission energy exceeds this threshold, the CU will drop the transmission.

## 3. Optimal Mode Decision Policy Based POMDP

In this study, we obtain an optimal mode decision policy by adopting POMDP for the object of maximizing the throughput of the CR system. Two operation modes, sleeping mode (S) and accessing mode (AC), are considered for the CU. As a normal device with limited energy resources, if the CU lacks energy for operations (i.e., spectrum sensing and transmission data), it will keep sleeping and only harvest energy for the next time operation. This operation is called sleeping mode. In the accessing mode, on the other hand, the CU performs spectrum sensing to detect the state of the PU and further if the state A of the PU is detected, the CU transmitter will send data to the CU receiver.

In spectrum sensing, consumed energy can significantly affect the throughput of the system, especially in the case of limited energy devices. Subsequently, in the next subsection we will propose an algorithm to obtain the optimal sensing energy for the CU.

### 3.1. Optimal Sensing Energy for Maximizing Throughput

The spectrum sensing of the CU, which is assumed to be performed by using an energy detection method, is to distinguish between two hypotheses of the PU, presence (P) or absence (A). Consider the Gaussian noise in the sensing channel, hence when the number of sensing samples *M* is relatively large (e.g., *M* > 200), the received signal energy *xE* can be closely approximated as a Gaussian random variable under both hypotheses such that [10]

(9) $$\begin{array}{c} xE\sim \{\begin{array}{cc} N\left(M,2M\right), & \text{A},\\ N\left(M\left(\gamma +1\right),2M\left(2\gamma +1\right)\right), & \text{P}, \end{array} \end{array}$$

where *γ* is the SNR of the sensing channel between the PU and the CU.

The decision about state of the PU can be made as follows:

(10) $$\begin{array}{c} G\left(t\right)=1,\;\text{if}\;xE\left(t\right)\geq \lambda ,\\ G\left(t\right)=0,\;\text{otherwise}, \end{array}$$

where *λ* is the energy threshold and “1” and “0” correspond to the states P and A of the PU, respectively.

The sensing performance of the CU can be evaluated by the probability of false alarm (*P*_*f*_) and the probability of detection (*P*_*d*_), which are given, respectively, as

(11) $$\begin{array}{c} P_{f}=Q\left(\frac{\lambda -M}{\sqrt{2M}}\right) \end{array}$$

and

(12) $$\begin{array}{c} P_{d}=Q\left(\frac{\lambda -M\left(\gamma +1\right)}{\sqrt{2M\left(2\gamma +1\right)}}\right). \end{array}$$

The number of sensing samples is assumed to be *M* = 2*τ*_*s*_ *ω*, where *τ*_*s*_ is the sensing time duration and *ω* is the bandwidth. Therefore, for the required probability of detection *P*_d_*, the probability of false alarm according to the sensing time *τ*_*s*_ can be calculated as follows:

(13) $$\begin{array}{c} P_{f}^{*}\left(\tau_{s}\right)=Q\left(\left(2\gamma +1\right)Q^{-1}\left(P_{d}^{*}\right)+\sqrt{\tau_{s}w}\gamma \right). \end{array}$$

Here, energy consumed by spectrum sensing is defined as *E*_*s*_. Then, we can assume that *E*_*s*_ is proportional to *τ*_*s*_ with a constant of proportionality *ε*; that is, *E*_*s*_ = *ετ*_*s*_. Therefore, the probability of false alarm depends on sensing energy according to

(14) $$\begin{array}{c} P_{f}^{*}\left(E_{s}\right)=Q\left(\left(2\gamma +1\right)Q^{-1}\left(P_{d}^{*}\right)+\sqrt{\frac{E_{s}w}{\varepsilon }}\gamma \right). \end{array}$$

If the sensing results of the CU is the state A of the PU, then the CU can transmit its data. But the throughput is achieved only when this transmission is performed and the PU is really in state A (i.e., the sensing result is correct). The average throughput according to sensing energy *E*_*s*_ can be defined as

(15) $$\begin{array}{c} R\left(E_{s}\right)=\frac{T-E_{s}/\varepsilon }{T}C_{0}\left(1-P_{f}^{*}\left(E_{s}\right)\right)\text{Pr}\left(H_{0}\right), \end{array}$$

where *T* is the total time frame for both spectrum sensing and data transmission and *C*_0_ is the standard throughput of the CR link, which is defined as *C*_0_ = log_2_(1 + SNR_CR_), where SNR_CR_ is the SNR received in the CU receiver.

The optimal value of *E*_*s*_ for each time frame such that the average throughput of the CU is maximized while maintaining a low level of interference with the PU (i.e., meet the requirement of *P*_d_*) can be found as the solution of an optimization problem as follows:

(16) $$\begin{array}{c} E_{s,\text{opt}}=\underset{E_{s}}{\arg}\;\max\left(\frac{T-E_{s}/\varepsilon }{T}C_{o}\left(1-P_{f}^{*}\left(E_{s}\right)\right)\text{Pr}\left(H_{0}\right)\right). \end{array}$$

**Figure 2:**
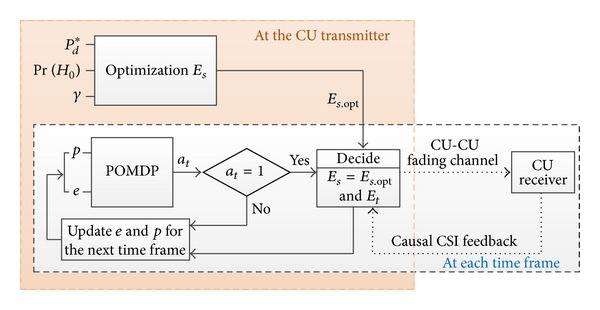
Flowchart of the proposed scheme.

The problem can be solved by using a numerical method and value of the optimal sensing energy *E*_*s*,opt_ will be utilized for the proposed optimal mode decision policy of the CU transmitter based on POMDP, as shown in Figure 2.

### 3.2. Optimal Mode Decision Policy

The optimal mode decision policy related to sleeping or accessing is formulated as the framework of POMDP. The value function *V*(*e*, *p*) is defined as the maximum total discounted throughput from the current time slot when the remaining energy is *e* and the belief regarding state A of the PU is *p*. The value function is given by

(17) $$\begin{array}{c} V\left(e,p\right)=\underset{a_{k},a_{k+1},\ldots }{\max}\;E\left\{\sum_{t=k}^{\infty }\alpha^{t-k}R\left(e_{t},p_{t},a_{t}\right)\;|\;e_{k}=e,p_{k}=p\right\}, \end{array}$$

where 0 ≤ *α* < 1 is the discount factor and *e*_*t*_ and *p*_*t*_ are the remaining energy and belief at the beginning of the *t*th time slot, respectively. *R*(*e*_*t*_, *p*_*t*_, *a*_*t*_) is the throughput of the CU achieved at the *t*th time slot, which is mainly dependent on *e*_*t*_, *p*_*t*_, and action *a*_*t*_. As described above, action can be either to remain sleeping or change to accessing; that is, *a*_*t*_ ∈ {*S*, AC}. If the CU decides to change to accessing mode it will use *E*_*s*,opt_ as sensing energy. In addition, an ATPC will calculate the transmission energy *E*_*t*_ according to the channel gain information which is provided by causal CSI feedback from the CU receiver.

#### 3.2.1. Sleeping Mode (*ϕ*_1_)

If the CU decides to remain sleeping, no throughput is achieved; then *R*(*e*_*t*_, *p*_*t*_, *S* | *ϕ*_1_) = 0 and the belief *p* for the next time slot is updated as follows:

(18) $$\begin{array}{c} p_{t+1}=p_{t}P_{\text{AA}}+\left(1-p_{t}\right)P_{\text{PA}}. \end{array}$$

Also, the remaining energy of the battery will be increased according to

(19) $$\begin{array}{c} e_{t+1}=e_{t}+E_{h}\left(t\right), \end{array}$$

with transition probability

(20) $$\begin{array}{c} \text{Pr}\left(e_{t}⟶e_{t+1}\;|\;\phi_{1}\right)=\text{Pr}\left[E_{h}\left(t\right)=e_{k}^{h}\right] \end{array}$$

for *k* = 1,2,…, *ζ*_*h*_.

#### 3.2.2. Accessing Mode

When the CU decides to change to accessing mode, the achieved throughput of the system depends on the observation of the CU. In this paper, we define 4 observations for the accessing mode of the CU which are as follows.

**Observation 1 (*ϕ*_2_)**

The sensing result is state P of the PU; then the CU does not transmit data and there will be no achieved throughput, *R*(*e*_*t*_, *p*_*t*_, AC∣*ϕ*_2_) = 0. The probability that *ϕ*_2_ happens is

(21) $$\begin{array}{c} \text{Pr}\left(\phi_{2}\right)=p_{t}P_{f}^{*}\left(E_{s,\text{opt}}\right)+\left(1-p_{t}\right)P_{d}^{*}. \end{array}$$

The belief *p* in the current time slot can be updated by using Bayes' rule as follows:

(22) $$\begin{array}{c} p_{t}^{u}=\frac{p_{t}P_{f}^{*}\left(E_{s,\text{opt}}\right)}{p_{t}P_{f}^{*}\left(E_{s,\text{opt}}\right)+\left(1-p_{t}\right)P_{d}^{*}}. \end{array}$$

As a result, the updated belief that the PU is in state A at the next time slot is given by

(23) $$\begin{array}{c} p_{t+1}=p_{t}^{u}P_{\text{AA}}+\left(1-p_{t}^{u}\right)P_{\text{PA}}. \end{array}$$

The updated remaining energy is obtained as:

(24) $$\begin{array}{c} e_{t+1}=e_{t}+E_{h}\left(t\right)-E_{s,\text{opt}} \end{array}$$

with transition probability

(25) $$\begin{array}{c} \text{Pr}\left(e_{t}⟶e_{t+1}\;|\;\phi_{2}\right)=\text{Pr}\left[E_{h}\left(t\right)=e_{k}^{h}\right] \end{array}$$

for *k* = 1,2,…, *ζ*_*h*_.

**Observation 2 (*ϕ*_3_)**

There is no PU signal detected (i.e., state A). The required transmission energy is smaller than the threshold th_*e*_*t*__; then the CU transmits data and can receive an ACK message. This means that the sensing result is correct (A is the real state of the PU) and the CU is successful at transmitting data. The throughput is achieved as

(26) $$\begin{array}{c} R\left(e_{t},p_{t},\mathrm{AC}\;|\;\phi_{3}\right)=\frac{T-E_{s,\text{opt}}/\varepsilon }{T}C_{0}. \end{array}$$

The probability that *ϕ*_3_ happens is

(27) $$\begin{array}{c} \text{Pr}\left(\phi_{3}\right)=\text{Pr}\left(E_{t}\left(t\right)\leq {\text{th}}_{e_{t}}\right)p_{t}\left(1-P_{f}^{*}\left(E_{s,\text{opt}}\right)\right). \end{array}$$

The belief and remaining energy for the next time slot can be updated, respectively, as

(28) $$\begin{array}{c} p_{t+1}=P_{\text{AA}}, \end{array}$$

(29) $$\begin{array}{c} e_{t+1}=e_{t}+E_{h}\left(t\right)-E_{s,\text{opt}}-E_{t}\left(t\right) \end{array}$$

with transition probability

(30) $$\begin{array}{c} \text{Pr}\left(e_{t}⟶e_{t+1}\;|\;\phi_{3}\right)=\text{Pr}\left[E_{h}\left(t\right)=e_{k}^{h}\right]\text{Pr}\left[E_{t}\left(t\right)=e_{m}^{t}\right] \end{array}$$

for all *k* = 1,2,…, *ζ*_*h*_ and *m* = 1,2,…, *L*_*g*_.

**Observation 3 (*ϕ*_4_)**

State A of the PU is detected. The required transmission energy is smaller than the threshold th_*e*_*t*__; then the CU transmits data but can not receive the ACK message. This means that the sensing result is incorrect (P is the real state of the PU), the transmission data fails, and *R*(*e*_*t*_, *p*_*t*_, AC∣*ϕ*_4_) = 0. The probability that *ϕ*_4_ is obtained is

(31) $$\begin{array}{c} \text{Pr}\left(\phi_{4}\right)=\text{Pr}\left(E_{t}\left(t\right)\leq {\text{th}}_{e_{t}}\right)\left(1-p_{t}\right)\left(1-P_{d}^{*}\right). \end{array}$$

The belief that the PU will be in state A at the next time slot is given as

(32) $$\begin{array}{c} p_{t+1}=P_{\text{PA}}. \end{array}$$

The remaining energy of the CU can be updated similar to the case of *ϕ*_3_.

**Observation 4 (*ϕ*_5_)**

The sensing result concludes that the PU is in state A. The required transmission energy exceeds the threshold th_*e*_*t*__; then the CU does not transmit data and *R*(*e*_*t*_, *p*_*t*_, AC∣*ϕ*_5_) = 0. The probability of the case *ϕ*_5_ is given as

(33) $$\begin{array}{c} \text{Pr}\left(\phi_{5}\right)=\text{Pr}\left(E_{t}\left(t\right)>{\text{th}}_{e_{t}}\right)P_{s,\text{A}}, \end{array}$$

where *P*_*s*,A_ is the probability that the sensing result is state A of the PU, which is given by

(34) $$\begin{array}{c} P_{s,\text{A}}=\left(p_{t}\left(1-P_{f}^{*}\left(E_{s,\text{opt}}\right)\right)+\left(1-p_{t}\right)\left(1-P_{d}^{*}\right)\right). \end{array}$$

Based on Observation 4, we can update the belief *p* of current time slot by using Bayes' rule as follows:

(35) $$\begin{array}{c} p_{t}^{u}=\frac{p_{t}\left(1-P_{f}^{*}\left(E_{s,\text{opt}}\right)\right)}{p_{t}\left(1-P_{f}^{*}\left(E_{s,\text{opt}}\right)\right)+\left(1-p_{t}\right)\left(1-P_{d}^{*}\right)}. \end{array}$$

Subsequently, the updated belief for the next time slot is calculated as

(36) $$\begin{array}{c} p_{t+1}=p_{t}^{u}P_{\text{AA}}+\left(1-p_{t}^{u}\right)P_{\text{PA}}. \end{array}$$

The updated remaining energy for the next time slot can be obtained similar to the case of *ϕ*_2_.

According to those observations, the value function in (17) can be expressed as follows:

(37) V(e,p)=maxak,ak+1,…{∑t=k∞αt−k∑ϕi∈atPr(ϕi)×∑et+1Pr(et⟶et+1 ∣ ϕi)×R(et,pt,at ∣ ϕi) ∣ ek=e,pk=p}.

The optimization problem in (37) can be solved to find an optimal mode decision for maximizing throughput of CR system by using the *value iterations* method [11].

## 4. Simulation Results

In this section, we present simulation results of the proposed scheme and the *Myopic* scheme that only considers the current time slot for the *value function* (i.e., *α* = 0) under the parameters as shown in Table 1.

**Figure 3:**
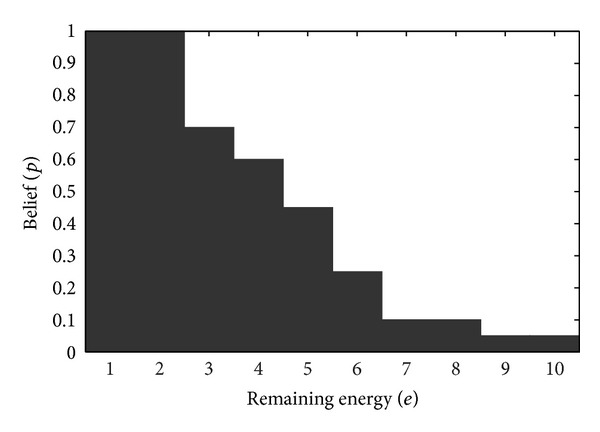
The optimal mode decision policy when *E*_ca_ = 10, *e*_mean_^*h*^ = 2, and *e*_mean_^*t*^ = 7 (black area: sleeping mode and white area: accessing mode).

Figure 3 shows the optimal mode decision policy for the sleeping and accessing modes based on the values of *p* and *e*. It can be seen that when the remaining energy is low, the CU changes to the accessing mode when value of *p* is high. In contrast, when the value of *p* is low, more remaining energy is required for carrying out the accessing mode.

**Figure 4:**
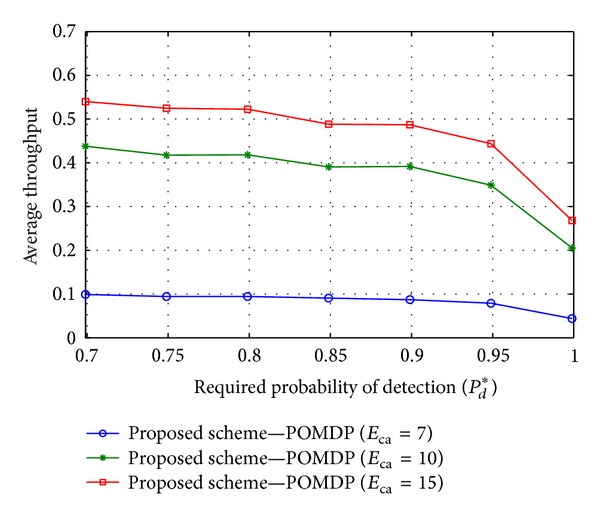
Average throughput versus the required detection probability *P*_d_* when *E*_ca_ = {7, 10, 15}, *e*_mean_^*t*^ = 7, and *e*_mean_^*h*^ = 2.

**Figure 5:**
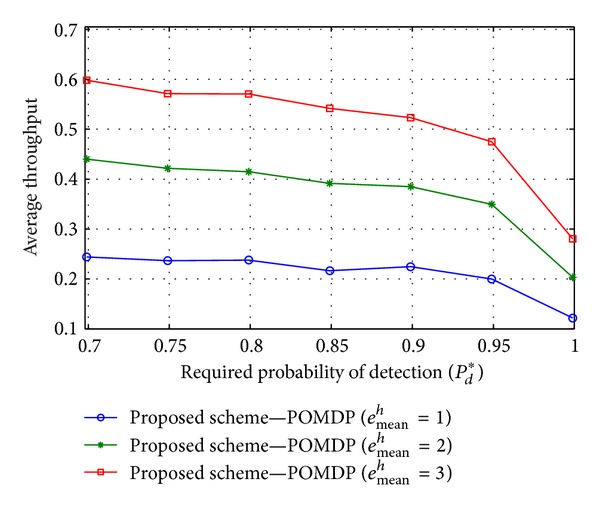
Average throughput versus the required detection probability *P*_d_* when *e*_mean_^*h*^ = {1, 2, 3}, *E*_ca_ = 10, and *e*_mean_^*t*^ = 7.

**Figure 6:**
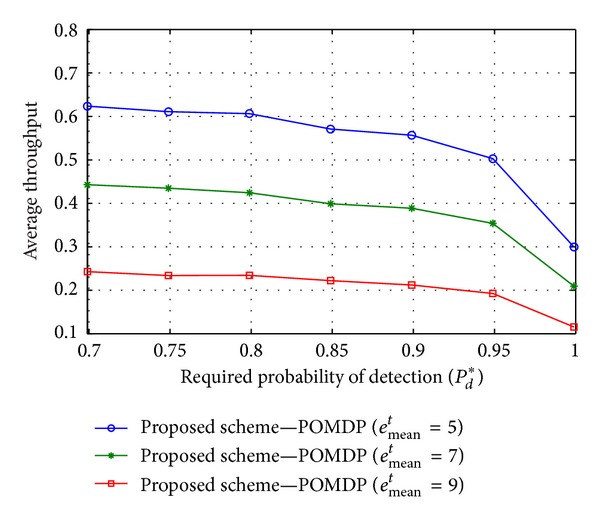
Average throughput versus the required detection probability *P*_d_* when *e*_mean_^*t*^ = {5, 7, 9}, *E*_ca_ = 10, and *e*_mean_^*h*^ = 2.

Figures 4, 5, and 6 illustrate average throughput according to the required probability of detection *P*_d_* in some cases of *E*_ca_, *e*_mean_^*h*^, and *e*_mean_^*t*^. It is clear that the required probability of detection *P*_d_* represents the protection level of the PU. That is, a high value of *P*_d_* offers a high protection level for the PU. However, the high protection level of the PU may reduce the opportunity for communication in the CR system. The clear relation between throughput and the value of *P*_d_* is shown through Figures 4, 5, and 6; that is, average throughput tends to decrease with the improvement in *P*_d_*. On the other hand, the increases in *E*_ca_ and *e*_mean_^*h*^ provide the CU with a higher probability of being active in accessing mode, which results in increase of average throughput. On the contrary, the higher transmission energy (i.e., higher *e*_mean_^*t*^) may reduce the average throughput in the case of a constrained energy resource.

**Figure 7:**
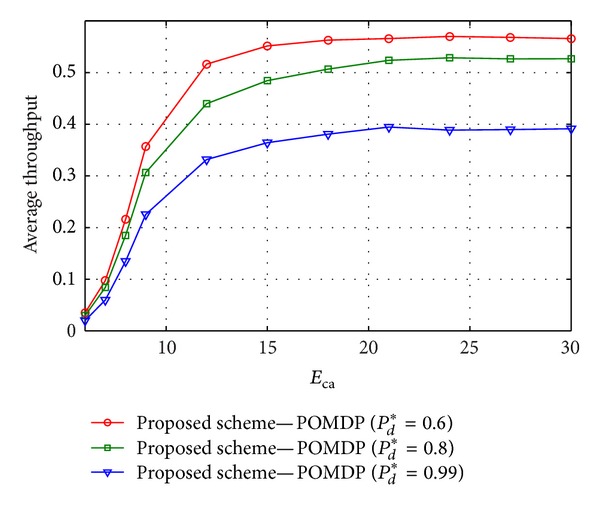
Average throughput versus the value of *E*_ca_ when the required probability of detection *P*_d_* = {0.6, 0.8, 0.99}, *e*_mean_^*t*^ = 7, and *e*_mean_^*h*^ = 2.

Figure 7 shows average throughput when the capacity of battery *E*_ca_ and the required values of *P*_d_* are considered. From the figure, it is observed that the average throughput increases with decreasing of *P*_d_* and the bigger capacity of the battery (i.e., bigger *E*_ca_) results in higher average throughput. However, when *E*_ca_ reaches a certain level that is sufficient to store all harvested energy, then the throughput can not be improved due to enhancement in *E*_ca_.

**Figure 8:**
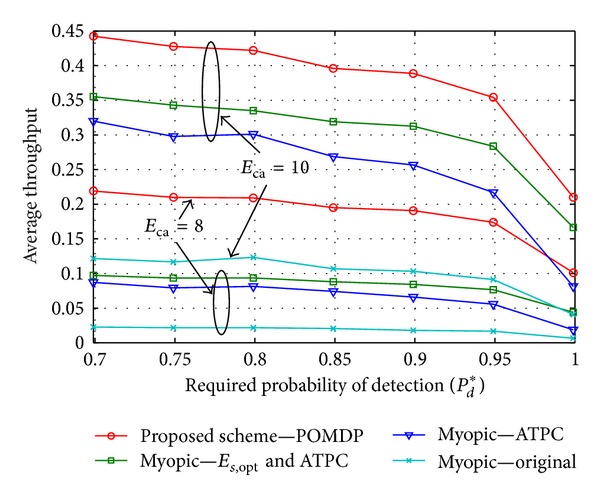
Average throughput of the proposed scheme in comparison with Myopic schemes for values of *E*_ca_ = {8, 10}, *e*_mean_^*t*^ = 7, and *e*_mean_^*h*^ = 2.

Figure 8 compares the POMDP-based proposed scheme with the Myopic scheme. We define three cases of the Myopic scheme in this simulation: (1) “Myopic-original,” the scheme is described in [5] in which no optimal sensing energy and no ATPC are considered; (2) “Myopic-ATPC,” Myopic scheme in which an ATPC is considered; (3) “Myopic-*E*_*s*,opt_ and ATPC,” Myopic scheme in which both optimal sensing energy *E*_*s*,opt_ and an ATPC are considered. It can be seen that an ATPC and/or an optimal sensing energy algorithm can improve throughput of the system compared with the “Myopic-original” scheme. In addition, the proposed scheme achieves better performance than all Myopic schemes because it considers the throughput of future time frame based on POMDP.

## 5. Conclusion

In this paper, a POMDP-based proposed scheme is investigated in order to find an optimal mode decision policy to maximize the throughput of the CR system. The random value of harvested energy considered in the proposed scheme is more practical than that in the previous studies. An ATPC scheme and an optimal sensing energy algorithm are proposed for efficient utilization energy from a limited capacity battery of the CU. Simulation results demonstrate that the proposed scheme significantly improves the throughput of the CR system. More specifically, throughput of the CR system depends on protection level of the PU system, *p*_d_*. With higher level of *p*_d_*, the opportunity for communication of CR system is decreased and corresponding throughput is also decreased. In addition, the increase of harvested energy and capacity of battery can improve throughput of the system. However, higher transmission energy reduces the throughput.

## Figures and Tables

**Table 1:** Simulation parameters.

Symbol	Description	Value
*γ*	SNR of the sensing channel	−10 dB
*T*	Total time frame	0.02 s
*f*_*s*_	Sensing bandwidth	0.2 × 10^6^ Hz
Pr (*H*_0_)	Average absence probability of the PU	0.9
*P*_AA_	Transition probability from state A to itself	0.8
*P*_PA_	Transition probability from state P to state A	0.1
*E*_ca_	Total capacity of battery	{7, 10, 15} units
*e*_mean_^*h*^	Mean value of harvested energy	{1, 2, 3} units
*e*_mean_^*t*^	Mean value of transmission energy	{5, 7, 9} units
